# Evaluation of self-stigma in patients with mental illness from hospitalization in the Mental Health Acute Unit

**DOI:** 10.1192/j.eurpsy.2023.941

**Published:** 2023-07-19

**Authors:** M. Valverde Barea, A. España Osuna, M. O. Solis Correa

**Affiliations:** Hospital Universitario Jaén, Jaén, Spain

## Abstract

**Introduction:**

Stigma is a complex process and a universal phenomenon that is part of all social groups and that is maintained by its functions related to the establishment of one’s own identity and the facilitation of socialization processes. The stigma of the patient is important to evaluate since it is a subjective experience that can have negative correlations in relation to self-esteem, empowerment and recovery orientation of the patient with mental illness. Hospitalization in mental health takes place at times of mental illness decompensation and is an intervention closely related to the stigma towards mental illness.

**Objectives:**

The objective of the study is to evaluate the stigma perceived by patients with mental illness hospitalized in an acute mental health unit.

**Methods:**

Observational study with 53 patients hospitalized in an acute mental health unit.

Variables collected: Sociodemographic variables (age, sex), clinical diagnosis and stigma is evaluated with the Illness Self-stigma Scale (ISMI).

**Results:**

Sample of 53 patients, 55% women and 44% men, the most frequent diagnoses among those admitted are psychosis spectrum 26.42%, depressive disorders 24.53%, personality disorders 22.64% and bipolar disorders 11.33%. The average age is 41.96 years, between 18 and 72 years. The self-stigma according to the scale (ISMI) we obtain as a total score the patient with the highest stigma scores 100 points and the one with the least scores 44 points. Regarding diagnoses, depressive disorders score 33-72 points, while psychotic disorders score 36-85 points. The highest scores in self-stigma in our study are in personality disorders 49-100 and borderline personality disorder stands out (100 points). In the 5 subscales such as alienation, self-stigma, perceived discrimination, social isolation and resistance to stigma. Higher scores in alienation stand out in all patients.

**Conclusions:**

Patients with personality disorders, especially borderline personality disorder, followed by psychotic disorders, present greater perceived self-stigma in our study than the rest of the patients; it is a very important factor that can affect the evolution of the clinical picture. This factor is important to establish the therapeutic plan and the different interventions, it would be recommended to assess the stigma together with the measures to reduce symptoms.

**Disclosure of Interest:**

None Declared

